# Exploring the association between theobromine intake and hepatic steatosis in young people

**DOI:** 10.1038/s41598-024-63863-6

**Published:** 2024-06-05

**Authors:** Yi Kong, Li Han, Zhongxin Zhu, Xingxing Chen

**Affiliations:** 1 Department of Pharmacy, The First People’s Hospital of Xiaoshan District, 311200 Hangzhou, Zhejiang People’s Republic of China; 2 Department of Comprehensive Ward, The First People’s Hospital of Xiaoshan District, Hangzhou, 311200 Zhejiang People’s Republic of China; 3 Clinical Research Center, The First People’s Hospital of Xiaoshan District, Xiaoshan Affiliated Hospital of Wenzhou Medical University, No. 199 South Shixin Road, Hangzhou, 311200 Zhejiang People’s Republic of China

**Keywords:** Controlled attenuation parameter, Hepatic steatosis, Fatty liver, Theobromine intake, NHANES, Non-alcoholic fatty liver disease, Disease prevention, Nutrition

## Abstract

The incidence of non-alcoholic fatty liver disease (NAFLD) tends to be younger. And the role of theobromine in fatty liver disease remains unclear. The purpose of this study was to investigate the relationship between dietary theobromine intake and degree of hepatic steatosis in individuals aged 45 and below, using data from the 2017–2020 National Health and Nutrition Examination Survey (NHANES) and liver ultrasonography transient elastography. A total of 1796 participants aged below 45 years were included from NHANES 2017–2020 data after applying exclusion criteria. Multivariate regression and subgroup analyses were conducted to examine the associations between theobromine intake and controlled attenuation parameter (CAP), adjusting for potential confounders. Generalized additive models and two-piecewise linear regression were used to analyze nonlinear relationships. In the unadjusted Model 1 and preliminarily adjusted Model 2, there was no significant correlation between theobromine intake and CAP values. However, in Models 3 and 4, which accounted for confounding factors, a higher intake of theobromine was significantly associated with lower CAP values. Subgroup analyses in the fully adjusted Model 4 revealed a significant negative correlation among individuals aged 18–45, women, and white populations. Nonlinear analysis revealed a U-shaped relationship in black Americans, with the lowest CAP values at 44.5 mg/day theobromine. This study provides evidence that higher theobromine intake is correlated with lower degree of hepatic steatosis in young people, especially those aged 18–45 years, women, and whites. For black Americans, maintaining theobromine intake around 44.5 mg/day may help minimize liver steatosis. These findings may help personalize clinical nutritional guidance, prevent the degree of hepatic steatosis, and provide pharmacological approaches to reverse fatty liver disease in young people.

## Introduction

The increasing occurrence of non-communicable diseases in the last thirty years has reshaped global health priorities, influenced by lifestyle shifts^1^ . The surge in non-alcoholic fatty liver disease (NAFLD) is connected to a new epidemic of chronic liver disease, which aligns with the worldwide escalation of obesity^2^. As obesity starts impacting individuals at a younger age, there is a rising incidence of NAFLD among the youth^3–5^. Epidemiological data indicate that while the global prevalence of fatty liver in children and adolescents is estimated at 8% to 20%^6^, this figure is likely underestimated due to diagnostic challenges in this age group^7^. In the United States, approximately 24% of young adults are impacted, a likely underestimated statistic^8^. Hence, early intervention and preventive strategies are crucial for addressing fatty liver in the young population.

Losing weight, whether through bariatric surgery or self-imposed low-calorie diet, can successfully enhance liver insulin sensitivity and treat NAFLD^9^. But in fact, weight loss is challenging, and sustaining this weight loss proves even more difficult^10,11^. Studies show that only 20% of obese individuals successfully maintain their weight loss^12^. Increased accumulation of fat in the form of triglycerides in hepatocytes is known as hepatic steatosis, which can progress to cirrhosis and liver failure. Consequently, researchers are actively seeking pharmaceutical methods to reverse hepatic steatosis.

Theobromine, primarily accumulated in cacao plants, is an important methylxanthine known for its stimulant effects and mild diuretic properties^13^. Recent studies indicate that theobromine may play a role in glucose metabolism, particularly by stimulating the pancreas to release more insulin and the liver to release more glucose, suggesting a potential impact on liver function^14^. Additionally, theobromine's subtle psychoactive effects and its influence on cardiovascular gene expression highlight its systemic impact, potentially extending to liver function^15^. However, the role of theobromine in fatty liver disease remains unclear. The latest National Health and Nutrition Examination Survey (NHANES) includes liver ultrasonography transient elastography using the controlled attenuation parameter (CAP) for diagnosing hepatic steatosis. The aim of this study is to investigate the relationship between dietary theobromine intake and degree of hepatic steatosis in individuals aged 45 and below, using data from the 2017–2020 NHANES. This study hopes to provide practical guidance for the prevention and management of fatty liver disease in young individuals.

## Materials and methods

### Statement of ethics

All participants gave their informed agreement, and the project was authorized by the National Center for Health Statistics Research Ethics Review Board.

### Study population

**Figure 1:**
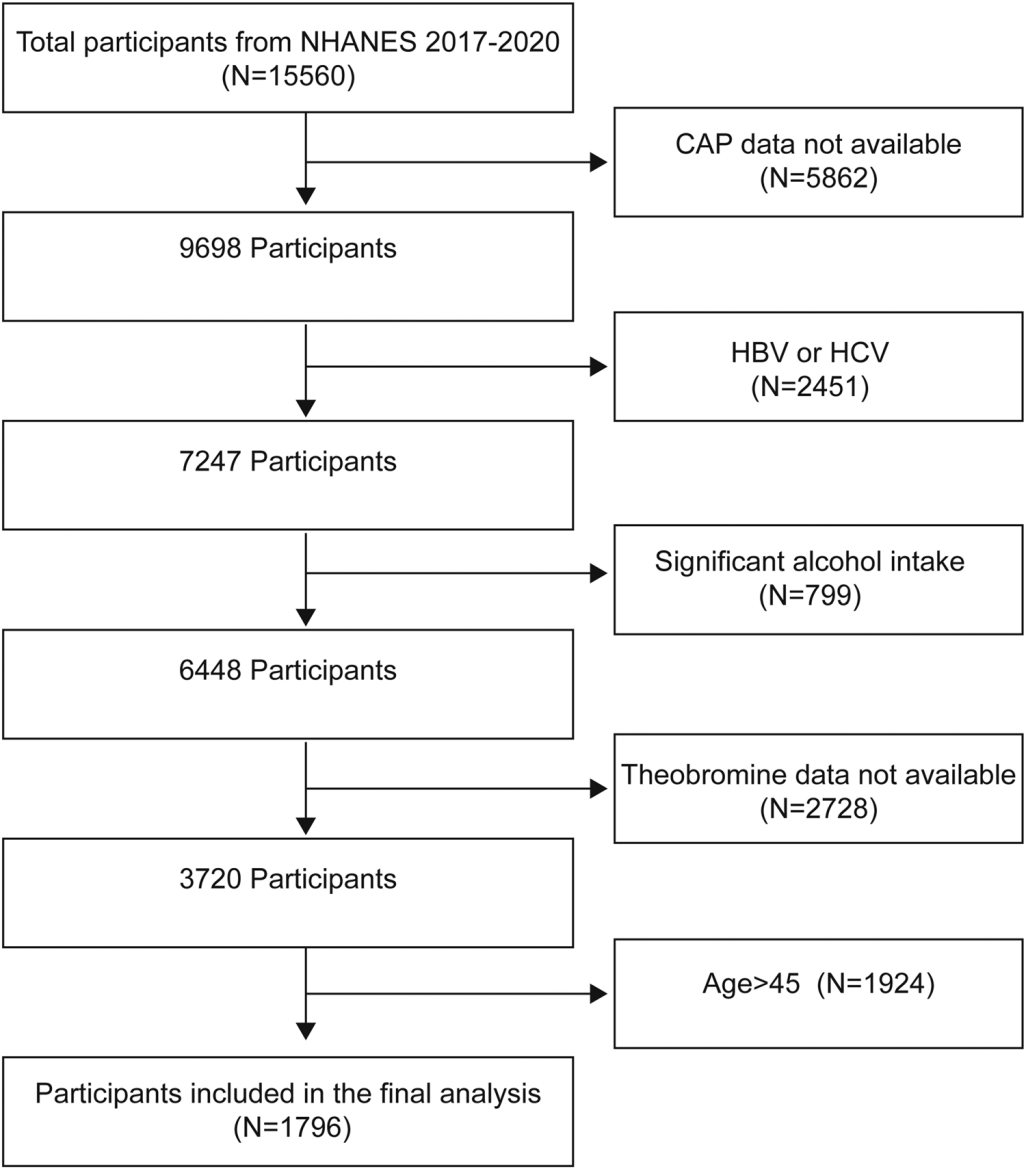
Flowchart of participant selection. *NHANES* National Health and Nutrition Examination Survey, *CAP* controlled attenuation parameter.

In order to achieve nationwide representation, NHANES, a thorough and ongoing cross-sectional national survey in the US, uses a stratified, multistage, clustered random sampling to collect diet and health data from the whole population^16^. Out of 15,560 participants in the 2017–2020 NHANES cycle, 9698 had available CAP data. We excluded 2451 participants testing positive for hepatitis B antigen, hepatitis C antibody, or hepatitis C RNA, 799 with significant alcohol consumption (4, 5, or more drinks daily), 2728 lacking theobromine intake data, and 1924 participants age > 45 years old. Ultimately, 1796 participants (ranging in age from 12 to 45 years) were included in the study. Figure 1 illustrates the sample selection flowchart.

### Variables

The investigation focused on dietary theobromine intake as the exposure factor. Theobromine intake assessment involved two 24-h food recall interviews. Three to ten days after the first interview, which took place at a mobile exam facility, there was a telephone interview. The 24-h dietary questionnaire collected the type and quantity of all beverages and foods (including foods such as chocolate) in the 24 h prior to the interview. The United States Department of Agriculture's Food and Nutrient Database for Dietary Studies was the source of information on nutrient intakes, including theobromine intake^17^. More details are available at http://www.ars.usda.gov/ba/bhnrc/fsrg. Theobromine intake per participant was averaged from two days of dietary recall data when available, or based on a single day's data otherwise (among all, 1654(92%) participants completed both the 24-h recalls).

The study's outcome variable, CAP, was measured using the FibroScan^®^ 502 V2 Touch equipped with liver ultrasonography transient elastography. This device records CAP by measuring ultrasonic attenuation, which reflects hepatic steatosis and indicates liver fatness. According to a recent key study, there is 90% sensitivity in detecting different degrees of hepatic steatosis when CAP values ≥ 274 dB/m, which indicate NAFLD status, are present^18^. This study, which is based on three earlier investigations, classifies CAP ≥ 302 dB/m as a sign of severe steatosis in instances of NAFLD^19–21^.

Our study incorporated categorical covariates such as gender, race/ethnicity, education level, smoking status, hypertension, diabetes, and cholesterol levels. Continuous covariates in our analysis included age, body mass index (BMI), alanine aminotransferase (ALT), aspartate aminotransferase (AST), γ-glutamyl transpeptidase (GGT), serum creatinine, serum albumin, and uric acid. Detailed data on dietary theobromine intake, CAP, and other variables are publicly accessible at http://www.cdc.gov/nchs/nhanes/.

### Statistical analysis

We took high volatility in our data set into account by using a weighted variance estimation technique. A weighted multivariate logistic regression model was employed to examine the correlation between theobromine intake and CAP (Model 1: no covariates were adjusted; Model 2: age, gender, and race/ethnicity were adjusted; Model 3: age, gender, race/ethnicity, education level, body mass index, smoking status, and the existence of diabetes, hypertension, and high cholesterol level were adjusted; Model 4: age, gender, race/ethnicity, education level, body mass index, smoking status, and the existence of diabetes, hypertension, and high cholesterol level, aspartate aminotransferase, alanine aminotransferase, γ- glutamyl transpeptidase, serum albumin, serum creatinine and uric acid were adjusted). The weighted χ2 test was utilized for categorical data in order to assess group differences, and the weighted linear regression model was applied for continuous variables. Subgroup analysis was performed using stratified multivariate regression analysis. Subgroups were divided according to age, sex, and race/ethnicity. The continuous variable age was divided into two groups (< 18 years; 18–45 years). Using generalized additive models and smooth curve fits, the nonlinear relationship between theobromine consumption and CAP was investigated. After finding nonlinearity, we used a recursive technique to calculate the inflection point in the theobromine intake and CAP connection. We next used a two-piecewise linear regression model to both sides of this point. All analyses were conducted using R (http://www.Rproject.org) and EmpowerStats (http://www.empowerstats.com), with a *P* value < 0.05 considered statistically significant.

### Ethics approval and consent to participate

The studies involving human participants were reviewed and approved by CDC’s National Center for Health Statistics Institutional Research Ethics Review Board. The patients/participants provided their written informed agreement to participate in this study. All our methods followed the guidelines of the Helsinki Declaration. And secondary analysis does not require additional institutional review committee approval.

## Results

There were 1796 participants in our study. The clinical features of CAP participants are shown in Table 1, which is arranged in columns for stratification. The severe steatosis group is more likely to be older, predominately male, and non-Hispanic White than the non-NAFLD group. Clinical factors including BMI, prevalence of diabetes, hypertension and high cholesterol also trended upwards with more advanced disease. Biochemical markers AST, ALT and GGT exhibited rising levels corresponding to CAP scores. Lifestyle patterns showed higher smoking rates and lower theobromine intake in severe steatosis.

**Table 1:** Weighted characteristics of the study population based on controlled attenuated parameter (CAP).

	Non-NAFLD (CAP < 274, n = 1299)	NAFLD (274 ≤ CAP < 302, n = 207)	Severe steatosis (CAP ≥ 302, n = 290)	*P* value
Age (years)	24.34 ± 9.82	29.46 ± 10.30	31.41 ± 9.78	< 0.0001
Gender (%)				< 0.0001
Men	44.37	53.07	57.68	
Women	55.63	46.93	42.32	
Race/Ethnicity (%)				< 0.0001
Mexican American	10.27	22.49	19.63	
Other Hispanic	7.45	5.95	9.91	
Non-Hispanic White	56.96	44.12	48.65	
Non-Hispanic Black	13.15	14.18	8.19	
Other Race	12.17	13.26	13.62	
Education level (%)				0.0072
Less than high school	8.35	9.47	10.93	
High school	22.46	24.08	34.15	
More than high school	69.19	66.45	54.94	
Income to poverty ratio	2.98 ± 1.67	2.79 ± 1.61	2.66 ± 1.54	0.0116
BMI (kg/m^2^)	24.79 ± 5.64	32.69 ± 6.34	36.61 ± 8.23	< 0.0001
Smoking status (%)				< 0.0001
Current	14.19	9.17	17.12	
Former	16.04	19.96	27.12	
Never	69.77	70.87	55.75	
Diabetes (%)				< 0.0001
Yes	0.83	2.01	6.17	
No	98.53	94.75	89.04	
Borderline	0.64	3.24	4.79	
Hypertension (%)	7.85	15.71	18.78	< 0.0001
High cholesterol level (%)	13.30	15.31	19.11	0.0458
AST (IU/L)	19.50 ± 10.24	21.14 ± 8.68	23.96 ± 16.62	< 0.0001
ALT (IU/L)	16.43 ± 9.09	25.07 ± 18.39	31.81 ± 22.19	< 0.0001
GGT (IU/L)	16.94 ± 13.57	28.38 ± 26.14	41.30 ± 47.66	< 0.0001
Serum albumin (g/L)	42.48 ± 3.15	40.98 ± 3.09	40.42 ± 3.46	< 0.0001
Serum creatinine (mg/dl)	0.77 ± 0.17	0.83 ± 0.25	0.80 ± 0.19	0.0002
Uric acid (mg/dl)	4.86 ± 1.18	5.32 ± 1.29	5.73 ± 1.56	< 0.0001
Theobromine intake (mg/day)	54.59 ± 69.22	59.88 ± 77.74	45.48 ± 51.42	0.0365

Mean ± SD for continuous variables: the *P* value was calculated by the weighted linear regression model. (%) for categorical variables: the *P* value was calculated by the weighted chi-square test.

The multivariate regression analysis's findings are presented in Table 2. In the unadjusted Model 1, daily theobromine intake showed no significant association with CAP (β =  − 0.03, 95% CI − 0.07, 0.01, *P* = 0.2063). In Model 2, which preliminarily adjusted for age, gender, and race/ethnicity, the association between theobromine intake and CAP was also not significant (β = 0.00, 95% CI − 0.04, 0.04, P = 0.9876). However, the Model 3, which further adjusted for education level, body mass index, smoking status, and the presence of diabetes, hypertension, and high cholesterol, revealed a statistically significant inverse association (β =  − 0.06, 95% CI − 0.11, − 0.01, *P* = 0.0208). When examining theobromine intake by quartiles, the results from Model 3 showed that individuals in the highest quartile (Q4) had a CAP value that was 6.59 dB/m lower compared to the reference group (Q1), demonstrating a significant linear trend (P for trend = 0.045). Even after accounting for various liver function and metabolic markers, Model 4's negative connection persisted (β =  − 0.06, 95% CI − 0.11, − 0.01, *P* = 0.0265). Analysis by quartiles of theobromine intake in Model 4 showed that CAP value of Q4 is 7.39 dB/m lower than Q1, demonstrating a significant linear trend (P for trend = 0.041). Based on subgroup analyses by age, gender, and race/ethnicity in Table 2, the relationship between theobromine intake and CAP was not evident in Model 1 and Model 2. However, further adjustments in Model 3 revealed a statistically significant negative correlation between theobromine intake and CAP among individuals aged 18–45 years (β =  − 0.05, 95% CI − 0.10, − 0.00, *P* = 0.0437), women (β =  − 0.08, 95% CI − 0.14, − 0.02, *P* = 0.0096), Whites (β =  − 0.09, 95% CI − 0.18, − 0.01, *P* = 0.0312), and other races (β =  − 0.13, 95% CI − 0.26, − 0.01, *P* = 0.0329). This negative correlation persisted in the fully adjusted Model 4 for individuals aged 18–45 years (β =  − 0.05, 95% CI − 0.10, − 0.00, *P* = 0.0419), women (β =  − 0.09, 95% CI − 0.15, − 0.02, *P* = 0.0081), and Whites (β =  − 0.09, 95% CI − 0.18, − 0.01, *P* = 0.0355).

**Table 2:** The association between theobromine intake (mg/day) and controlled attenuation parameter (dB/m).

	Model 1 β (95% CI) *P* value	Model 2 β (95% CI) *P* value	Model 3 β (95% CI) *P* value	Model 4 β (95% CI) *P* value
Theobromine intake (mg/day)	− 0.03 (− 0.07, 0.01) 0.2063	0.00 (− 0.04, 0.04) 0.9876	− 0.06 (− 0.11, − 0.01) 0.0208	− 0.06 (− 0.11, − 0.01) 0.0265
Theobromine intake quartile				
Q1 (0.5–12.5 mg/day)	Reference	Reference	Reference	Reference
Q2 (12.6–32.0 mg/day)	− 9.76 (− 17.93, − 1.59) 0.0193	− 2.63 (− 10.22, 4.95) 0.4960	2.73 (− 5.79, 11.25) 0.5303	2.55 (− 6.10, 11.19) 0.5640
Q3 (32.1–62.0 mg/day)	− 3.88 (− 12.09, 4.33) 0.3543	1.86 (− 5.73, 9.46) 0.6307	− 2.18 (− 10.53, 6.18) 0.6099	− 6.45 (− 15.03, 2.13) 0.1412
Q4 (62.1–556.0 mg/day)	− 8.33 (− 16.36, − 0.30) 0.0422	0.12 (− 7.36, 7.60) 0.9744	− 6.59 (− 15.14, 1.95) 0.1308	− 7.39 (− 16.03, 1.26) 0.0944
* P* for trend	0.043	0.795	0.045	0.041
Subgroup analysis stratified by age				
< 18 years	0.03 (− 0.01, 0.08) 0.1656	0.04 (− 0.01, 0.09) 0.1028	0.05 (− 0.01, 0.11) 0.0785	0.05 (− 0.01, 0.11) 0.0772
18–45 years	− 0.03 (− 0.09, 0.04) 0.4117	− 0.03 (− 0.09, 0.03) 0.3235	− 0.05 (− 0.10, − 0.00) 0.0437	− 0.05 (− 0.10, − 0.00) 0.0419
Subgroup analysis stratified by gender				
Men	− 0.03 (− 0.09, 0.03) 0.3233	0.00 (− 0.05, 0.06) 0.9192	− 0.01 (− 0.10, 0.07) 0.7532	0.00 (− 0.08, 0.09) 0.9105
Women	− 0.03 (− 0.09, 0.03) 0.3101	− 0.00 (− 0.06, 0.05) 0.9662	− 0.08 (− 0.14, − 0.02) 0.0096	− 0.09 (− 0.15, − 0.02) 0.0081
Subgroup analysis stratified by race/ethnicity				
Mexican American	0.03 (− 0.12, 0.18) 0.6602	0.08 (− 0.06, 0.22) 0.2565	− 0.05 (− 0.23, 0.13) 0.6075	− 0.09 (− 0.26, 0.08) 0.3080
Other Hispanic	− 0.06 (− 0.22, 0.10) 0.4469	− 0.07 (− 0.23, 0.08) 0.3582	0.00 (− 0.20, 0.21) 0.9727	0.00 (− 0.21, 0.22) 0.9681
Non-Hispanic White	− 0.03 (− 0.10, 0.04) 0.4095	− 0.00 (− 0.07, 0.06) 0.9540	− 0.09 (− 0.18, − 0.01) 0.0312	− 0.09 (− 0.18, − 0.01) 0.0355
Non-Hispanic Black	0.06 (− 0.03, 0.14) 0.2041	0.09 (0.00, 0.17) 0.0458	0.10 (0.01, 0.18) 0.0313	0.11 (0.02, 0.20) 0.0161
Other race	− 0.06 (− 0.15, 0.03) 0.2083	− 0.05 (− 0.14, 0.04) 0.2542	− 0.13 (− 0.26, − 0.01) 0.0329	− 0.12 (− 0.25, 0.01) 0.0830

Model 1: no covariates were adjusted.

Model 2: age, gender, and race/ethnicity were adjusted.

Model 3: age, gender, race/ethnicity, education level, body mass index, smoking status, and the existence of diabetes, hypertension, and high cholesterol level were adjusted.

Model 4: age, gender, race/ethnicity, education level, body mass index, smoking status, and the existence of diabetes, hypertension, and high cholesterol level, aspartate aminotransferase, alanine aminotransferase, γ- glutamyl transpeptidase, serum albumin, serum creatinine and uric acid were adjusted.

In the subgroup analysis stratified by age, gender and race/ethnicity, the model is not adjusted for age, gender and race/ethnicity, respectively.

**Figure 2:**
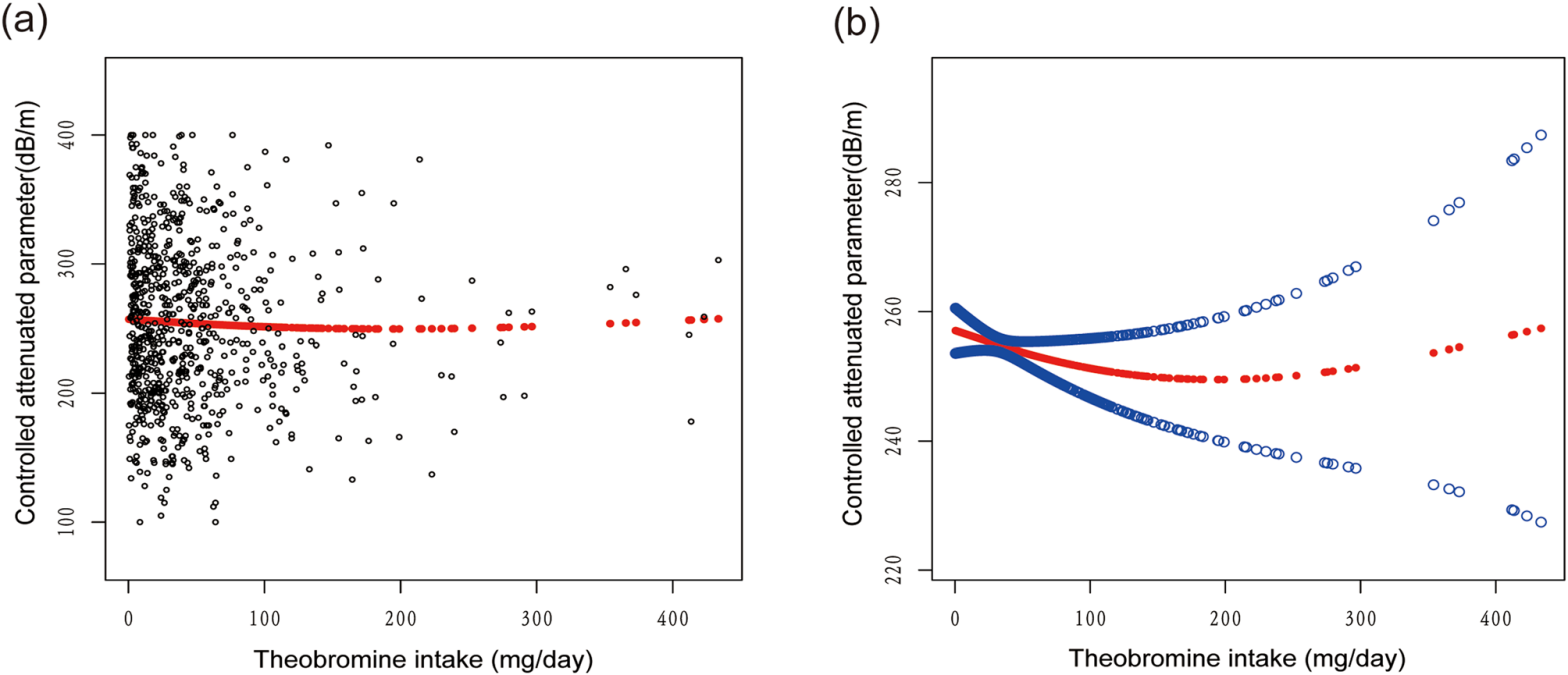
The association between theobromine intake and controlled attenuation parameter. (**a**) Each black point represents a sample. (**b**) Solid rad line represents the smooth curve fit between variables. Blue bands represent the 95% of confidence interval from the fit. Age, gender, race/ethnicity, education level, body mass index, smoking status, and the existence of diabetes, hypertension, and high cholesterol level, aspartate aminotransferase, alanine aminotransferase, γ- glutamyl transpeptidase, serum albumin, serum creatinine and uric acid were adjusted.

**Figure 3:**
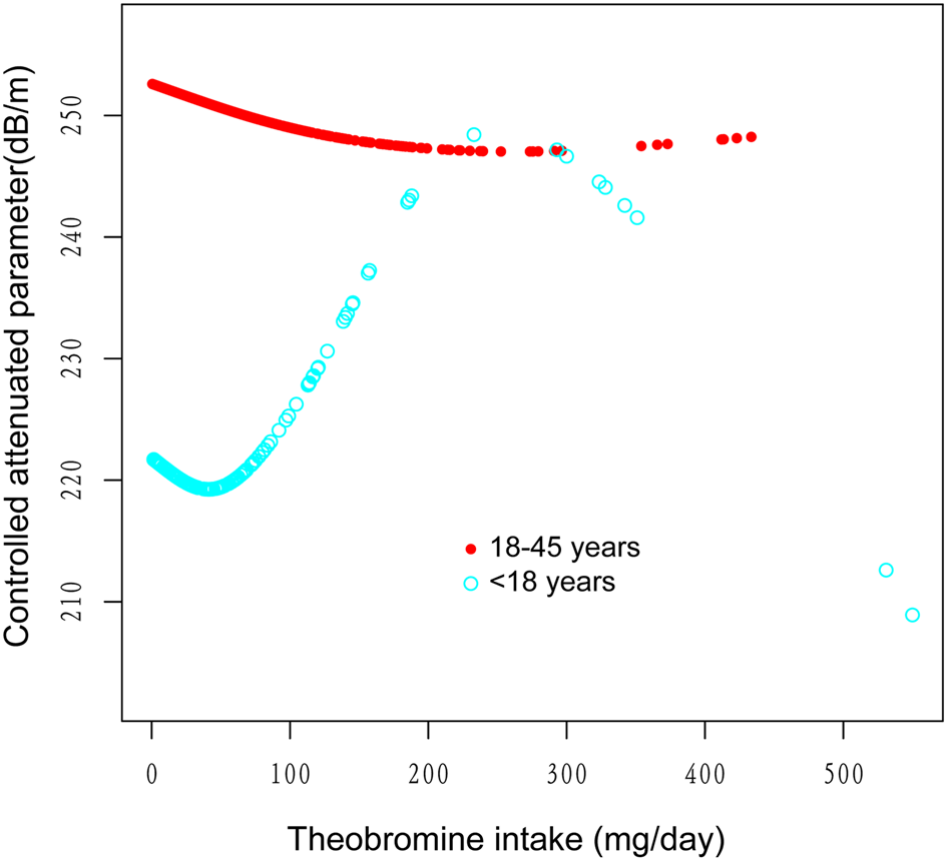
The association between theobromine intake and controlled attenuation parameter stratified by age. Gender, race/ethnicity, education level, body mass index, smoking status, and the existence of diabetes, hypertension, and high cholesterol level, aspartate aminotransferase, alanine aminotransferase, γ- glutamyl transpeptidase, serum albumin, serum creatinine and uric acid were adjusted.

**Figure 4:**
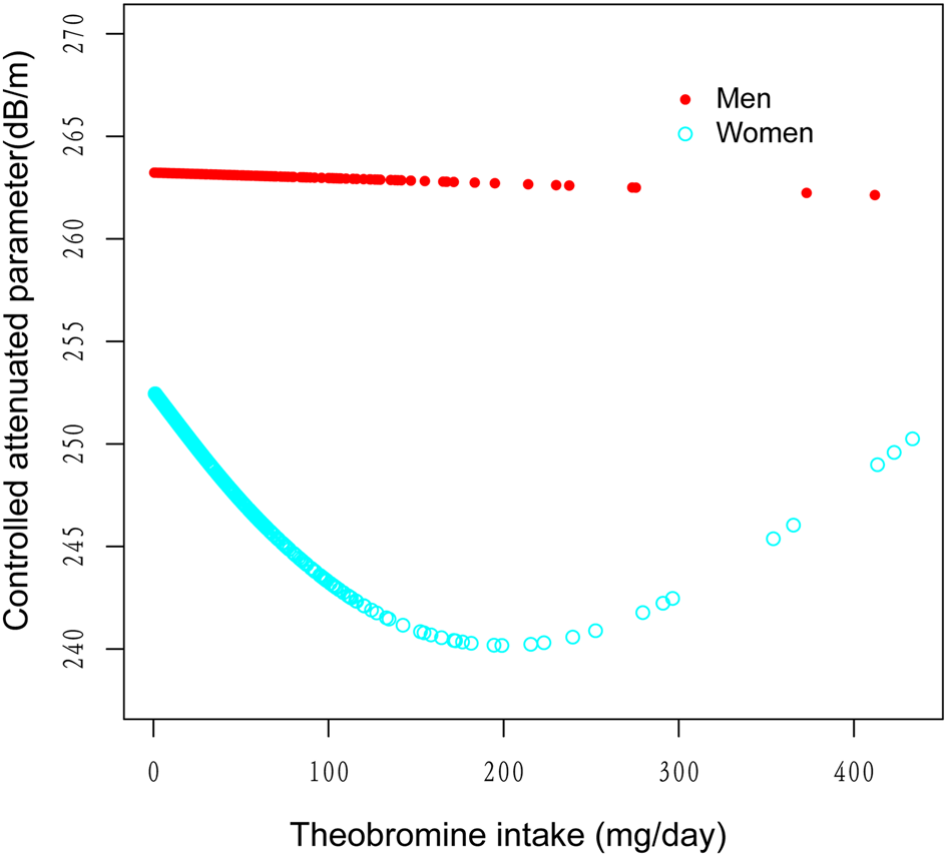
The association between theobromine intake and controlled attenuation parameter stratified by gender. Age, race/ethnicity, education level, body mass index, smoking status, and the existence of diabetes, hypertension, and high cholesterol level, aspartate aminotransferase, alanine aminotransferase, γ- glutamyl transpeptidase, serum albumin, serum creatinine and uric acid were adjusted.

**Figure 5:**
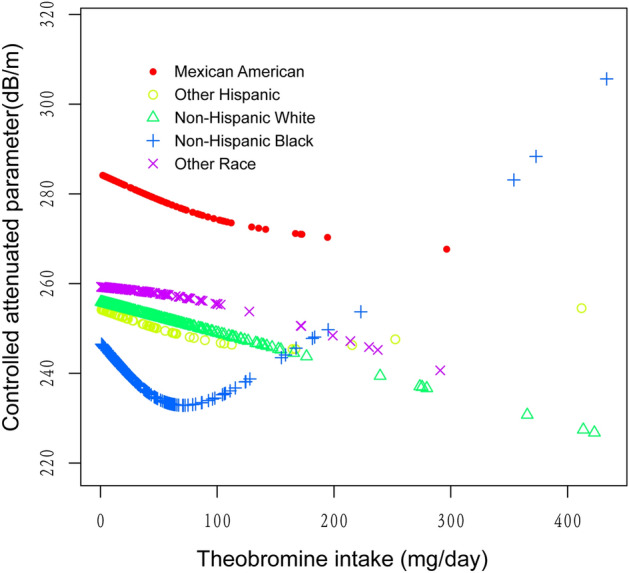
The association between theobromine intake and controlled attenuation parameter stratified by race/ethnicity. Age, gender, education level, body mass index, smoking status, and the existence of diabetes, hypertension, and high cholesterol level, aspartate aminotransferase, alanine aminotransferase, γ- glutamyl transpeptidase, serum albumin, serum creatinine and uric acid were adjusted.

The generalized additive models and smooth curve fits that were utilized to explain the nonlinear relationship between theobromine consumption and CAP are shown in Figs. 2, 3, 4 and 5. We found a nonlinear relationship between theobroline intake and CAP among blacks and performed a further threshold effect analysis. Using a two-piecewise linear regression model, the point of inflection for the U-shaped relationship between theobromine intake and CAP in black populations was determined to be 44.5 mg/day (Table 3). Below this threshold, a statistically significant inverse association was found (β =  − 0.58, 95% CI − 1.02, − 0.14, *P* = 0.0114). However, above 44.5 mg/day intake, the association reversed to a statistically significant positive relationship (β = 0.21, 95% CI 0.10, 0.31, *P* = 0.0002). The likelihood ratio test comparing the piecewise model to the standard linear model was statistically significant (*P* = 0.001), indicating the piecewise model provided a better fit to the data.

**Table 3:** Threshold effect analysis of theobromine intake on controlled attenuation parameter in non-Hispanic Black using the two-piecewise linear regression model.

controlled attenuation parameter	Adjusted β (95% CI), *P* value
Non-Hispanic Black	
Fitting by the standard linear model	0.11 (0.02, 0.20) 0.0161
Fitting by the two-piecewise linear model	
Inflection point	44.5
Theobromine < 44.5 (mg/day)	− 0.58 (− 1.02, − 0.14) 0.0114
Theobromine > 44.5 (mg/day)	0.21 (0.10, 0.31) 0.0002
Log likelihood ratio	0.001

Age, gender, education level, body mass index, smoking status, and the existence of diabetes, hypertension, and high cholesterol level, aspartate aminotransferase, alanine aminotransferase, γ- glutamyl transpeptidase, serum albumin, serum creatinine and uric acid were adjusted.

## Discussion

This research utilized NHANES data collected in the United States between 2017 and 2020. It explored the correlation between theobromine consumption and liver steatosis among 1796 participants aged below 45. Adjusted for potential confounders, multifactorial regression analysis showed an inverse relationship between daily theobromine intake and CAP values. This indicates that higher levels of theobromine intake correlate with reduced liver steatosis. After subgroup analysis, we found that this correlation was especially pronounced in people aged 18–45 years, women and white groups. Our results align with those from animal model studies. Mouse experiments indicated that theobromine treatment notably decreased liver steatosis, reduced lipid accumulation, lowered blood lipid levels, and enhanced insulin sensitivity^22,23^. This study extends these findings to a wider human demographic, suggesting potential therapeutic approaches for hepatic steatosis in young people, especially those aged 18–45 years, women, and whites.

The mechanism by which theobromine intake may alleviate fatty liver involves its main components, like pyruvic acid, mimicking the action of fibroblast growth factor 21 (FGF21) and activating the FGF21 signaling pathway^24^. This regulation affects energy metabolism and mitochondrial function related to fatty liver. Additionally, theobromine influences the expression of transcription factors related to adipogenesis, such as peroxisome proliferator-activated receptor γ (PPAR γ) and CCAAT/enhancer binding proteins (C/EBPα)^25^. It effectively inhibits the early differentiation of fat cells, thereby reducing liver fat formation.

Moreover, nonlinear analysis of the data revealed a U-shaped relationship between theobromine intake and CAP values in black Americans under 45 years. A critical point was identified at 44.5 mg/day. Intake below this threshold was inversely related to CAP values, while intake above it showed a positive correlation. This suggests that for young black Americans, maintaining theobromine intake at this threshold minimizes liver steatosis and maximizes liver protection. In daily life, theobromine is primarily derived from cocoa products such as chocolate and hot cocoa drinks^26^. Dark chocolate and pure cocoa blocks contain higher concentrations of theobromine^27^. For instance, 1 g of dark chocolate typically contains around 1–2 mg of theobromine^27^. To achieve the 44.5 mg of theobromine recommended in our study, approximately 22.25 to 44.5 g of dark chocolate per day would be required (specific intake can be calculated according to product labels).

To our knowledge, this is the first study to demonstrate such a specific relationship in black populations. The differences observed between racial groups could be attributed to genetic risks, lifestyle habits, and other factors^28^. However, more prospective studies with considerable sample sizes are needed for further validation. Our study's cohort size strengthens its findings, as NHANES aims to produce nationally representative estimates.

Nevertheless, there are limitations. First, transient elastography (CAP) was used to characterize hepatic steatosis instead of biopsy, which may introduce bias into the assessment process. Additionally, data on theobromine intake calculated based on 24-h dietary recall may be subject to reporting bias and exposure assessment bias; self-reported confounding factors could also be influenced by this bias; some potential unmeasured variables in NHANES may lead to residual confounding bias. Finally, due to its cross-sectional design, the study’s conclusions are correlational, not causal.

## Conclusions

Our research indicates a negative correlation between theobromine intake and CAP values in most Americans under 45, especially those aged 18–45 years, women, and whites. In young black Americans, this relationship follows a U-shaped curve, with CAP values being lowest at the inflection point of theobromine levels. These findings provide valuable insights for clinical guidance on theobromine intake and nutrition plan design, potentially aiding in the prevention of liver steatosis in young populations. Focusing on theobromine research could result in the development of pharmaceutical treatments for liver steatosis reversal.

## Data Availability

All NHANES data for this study are publicly available and can be found here: https://wwwn.cdc.gov/nchs/nhanes.
